# Double-punching PMBL

**DOI:** 10.18632/oncotarget.2827

**Published:** 2014-12-01

**Authors:** Jochen K. Lennerz

**Affiliations:** Department of Pathology, Massachusetts General Hospital/Harvard Medical School, Boston, MA, USA

Success stories in looking for effective strategies against cancer often have their roots in pathology. In case of primary mediastinal B-cell lymphoma (PMBL), this was likely the recognition as a distinct subtype of diffuse large B-cell lymphoma (DLBCL) in the early 80s [1, 2]. Initially a controversial entity, over the last 30 years, it has become clear that PMBL differs in pathogenesis, clinical presentation and clinical course. PMBL is thought to arise from thymic medullary B cells, has a high incidence among young women, and is characterized by aggressive local invasion. With chemoimmunotherapy (e.g. R-CHOP scheme), PMBL now carries a relatively good prognosis and novel strikingly efficient treatment options have been reported [3]. Thus, PMBL has come full circle from recognition to effective oncologic management. While primary treatment failure is uncommon, when it occurs, it is often rapid and difficult to treat. Thus, evolving treatment paradigms now aim to incorporate an expanded understanding of the underlying molecular-genetic aberrations of this particular lymphoma [2].

PMBL is also genetically distinct and the presence of the anti-apoptotic and pro-proliferative factors BCL6 and pSTAT6 are two key molecular features of PMBL. Recently, combined targeting of BCL6 and activated STAT6 via specific chemical inhibitors resulted in additive efficacy regarding the negative effects on cell viability in PMBL [4]. Notably, the distribution of these factors in clonal cell lines was heterogenous with a distribution of BCL6 and STAT6 to mutually exclusive subsets of cells [4]. Given the partial dependence from each of these co-existent oncogenic factors, Häberle et al. in a recent issue of oncoscience [5], evaluated whether molecular targeting of each factor in combination with components of the current chemoimmunotherapy (R-CHOP) holds therapeutic potential.

Specifically, the authors combined si-RNA mediated knock-down of either BCL6 or STAT6 in all three currently available PMBL cell lines with subsequent treatment with doxorubicin, rituximab, or vincristine. Notably, the authors applied doses that had little to no effect by themselves; doxorubicin 0.1nM (IC50:100nm), vincristine 0.1nM (IC50:2.7nM), and rituxamib 1μg/ml (vs. typically applied at 10μg/ml). With some variability, the authors' report that in two of the three cells lines (K1106 and U2940), initial depletion of one subset of cells (i.e., BCL6 or STAT6), renders the remaining cells hypersensitive to the low-dose components of the chemoimmunotherapy (Figure 1). While the authors did not report whether dual knock-down of BCL6 and STAT6 had a similar (over even more pronounced) sensitizing effect and it remains to be determined whether the same sensitization occurs with the previously reported chemical inhibitors, the data by Häberle et al. [5] are important for several reasons:

**Figure 1:**
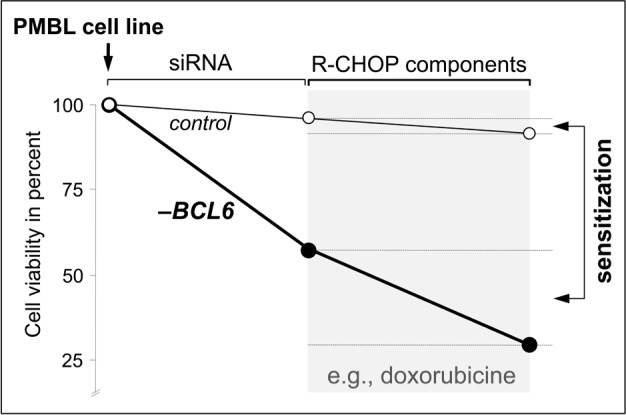
BCL6 knock-down induced chemosensitization in PMBL (data from [5])

First, in the current study the authors report a lack of sensitization in one of the PMBL cell lines that harbors STAT6 mutations (MedB-1). These ‘negative’ data underscore the importance of delineating the specific role of STAT6 mutations in PMBL. Actually, the same group described mutations in the DNA-binding domain of STAT6 in ~36% of PMBL cases [6]. Collectively these data suggest that STAT6 mutant cases may represent a distinct subset of PMBL cases – at least with respect to sensitization to chemotherapy using prior BCL6 and/ or STAT6 depletion. However, these notions remain speculative until the functional, clinicopathological or prognostic implications of STAT6 mutations in PMBL have been delineated.

Second, in combination with the prior report on intratumoral heterogeneity in PMBL, the current data underscore that targeting specific molecular aberrations deteriorates an apparent micro-organization between PMBL cells. Specifically, each cell line is composed of for example BCL6-positive and BCL6-negative subsets of cells. Now the group reports that si-RNA mediated elimination of part of the cellular population (e.g., BCL6) renders the remaining cells highly sensitive to components the R-CHOP regimen. This demonstration of an apparent intricate interplay between cells in a clonal population falls at first glance in line with several studies on intratumoral heterogeneity. However, demonstrating chemosensitization in a lymphoma provides an elegant and therapeutically relevant rational for a more in-depth understanding of the intratumoral architecture of PMBL. In fact, the demonstrated molecularly-targeted reprogramming –via elimination of one subset of cells– emphasizes that the cellular composition of a lymphoma may not be as monomorphous as previously assumed.

While the precise mechanism underlying the described sensitization remains to be determined, clinical trials employing JAK/STAT inhibitors are ongoing and solid preclinical data on novel BCL6 inhibitors are available [7]. Thus the study by Häberle et al. [5] is noteworthy because it delivers proof of principle that the combination of novel molecular strategies with established therapies may hold potential clinical value in PMBL.

## References

[1] Barth TF (2002). Lancet Oncol.

[2] Hamlin PA (2014). J Clin Oncol.

[3] Dunleavy K (2013). N Engl J Med.

[4] Ritz O (2013). Oncotarget.

[5] Häberle MT (2014). Oncoscience.

[6] Ritz O (2009). Blood.

[7] Cerchietti LC (2010). Cancer Cell.

